# Spatial and Temporal Distribution of Arabinogalactan Proteins during *Larix decidua* Mill. Male Gametophyte and Ovule Interaction

**DOI:** 10.3390/ijms22094298

**Published:** 2021-04-21

**Authors:** Katarzyna Rafińska, Katarzyna Niedojadło, Michał Świdziński, Janusz Niedojadło, Elżbieta Bednarska-Kozakiewicz

**Affiliations:** 1 Department of Environmental Chemistry and Bioanalytics, Faculty of Chemistry, Nicolaus Copernicus University, Gagarina 7, 87-100 Toruń, Poland; katraf@umk.pl; 2 Department of Cellular and Molecular Biology, Faculty of Biological and Veterinary Sciences, Nicolaus Copernicus University, Lwowska 1, 87-100 Toruń, Poland; michalswidzinski78@gmail.com (M.Ś.); janiaszn@umk.pl (J.N.); ebedn@umk.pl (E.B.-K.)

**Keywords:** gymnosperms, ArabinoGalactan Proteins (AGPs), pollen grain, pollen tube, ovule, extracellular matrix (ecm), progamic phase, fertilization

## Abstract

The role of ArabinoGalactan Proteins (AGPs) in the sexual reproduction of gymnosperms is not as well documented as that of angiosperms. In earlier studies, we demonstrated that AGPs play important roles during ovule differentiation in *Larix decidua* Mill. The presented results encouraged us to carry out further studies focused on the functions of these unique glycoproteins during pollen/pollen tube and ovule interactions in *Larix*. We identified and analyzed the localization of AGPs epitopes by JIM4, JIM8, JIM13 and LM2 antibodies (Abs) in male gametophytes and ovule tissue during pollination, the progamic phase, and after fertilization and in vitro growing pollen tubes. Our results indicated that (1) AGPs recognized by JIM4 Abs play an essential role in the interaction of male gametophytes and ovules because their appearance in ovule cells is induced by physical contact between reproductive partners; (2) after pollination, AGPs are secreted from the pollen cytoplasm into the pollen wall and contact the extracellular matrix of stigmatic tip cells followed by micropylar canal cells; (3) AGPs synthesized in nucellus cells before pollen grain germination are secreted during pollen tube growth into the extracellular matrix, where they can directly interact with male gametophytes; (4) in vitro cultured pollen tube AGPs labeled with LM2 Abs participate in the germination of pollen grain, while AGPs recognized by JIM8 Abs are essential for pollen tube tip growth.

## 1. Introduction

In seed plants, the extracellular matrix (ecm) of the female reproductive organs is the site of interactions with the male gametophyte, which begins during pollination and continues throughout the progamic phase. Many studies of model angiosperm plants have revealed the important role of pectins (HomoGalacturonans, HGs) and ArabinoGalactan Proteins (AGPs) in these processes [1,2,3,4]. The interaction results in a change in the activity of the wall enzymes of both reproductive partners, leading to alterations in the composition of the extracellular matrix (ecm), including the level of Ca^2+^ ions relevant for germination, pollen tube growth and double fertilization [5,6,7]. Given their unique structural properties, such as the diversity of the core protein, GPI (glycosylphosphatidylinositol) anchor and heterogeneity of the glycan chains, the large family of AGPs represents crucial candidates in these processes [8,9,10]. First, AGPs affect the properties of the cell wall. By interacting with β-glycans, AGPs control cell wall rigidity and extensibility. Type II AGPs may act as crosslinkers and bind with HGs chains or other cell wall polysaccharides, which strengthens the cell wall structure [9,11,12,13,14,15]. On the other hand, a small amount of AGPs incorporated into the pectin gel can cause a significant increase in its porosity [16]. The composition and plasticity of the cell wall are very important for tip-growing cells, including pollen tubes [17,18,19,20,21]. Moreover, GPI-anchored AGPs link the plasma membrane with the cytoskeleton and are involved in controlling cell shape by influencing cortical microtubule orientation and actin polymerization [22,23,24]. Second, AGPs can also bind Ca^2+^ ions and serve as extracellular calcium capacitors. Thus, AGPs are involved in calcium-mediated signaling pathways and create an environment for ovule development, pollen tube growth and guidance, fertilization and polytubey block [15,25,26,27]. Third, AGPs may act as signaling molecules participating in numerous reproductive processes. GPI-anchored AGPs can be released to the extracellular matrix through specific phospholipases and are involved in cell-to-cell interactions [9,28,29]. Additionally, specific sugar chains cleaved from AGPs by specific endogluconases may act as signals that move long distances and as morphogens and nutrients [30,31]. AGPs act as chemoattractants for growing pollen tubes [32,33,34,35].

**Figure 1:**
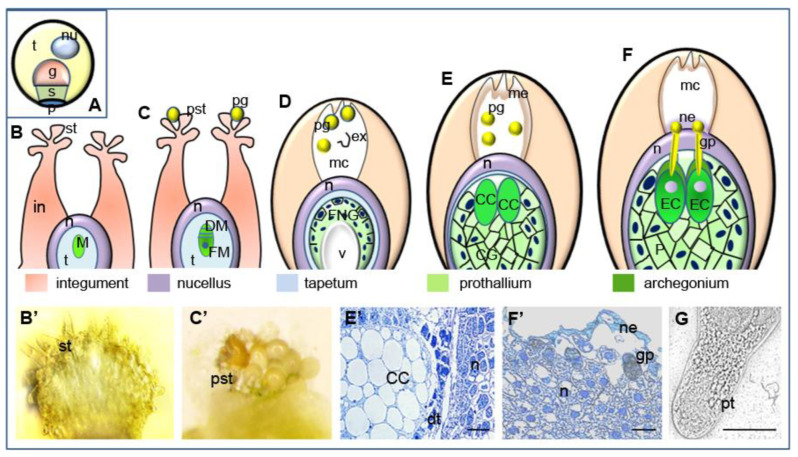
Successive stages of pollen-ovule interaction in *L. decidua*. (**A**) Mature pollen grain containing tube cell, generative cell, sterile cell and two degenerated prothallial cells; (**B**) unpollinated ovule (megasporocyte stage), (**B’**) unpollinated stigmatic tip, (**C**) pollinated ovule (stage of megaspores), (**C’**) stigmatic tip with pollen grains, (**D**) free nuclear gametophyte stage—pollen grains are engulfed into the micropylar canal and shed their exine, (**E**) cellular gametophyte stage—pollen grains are carried to the nucellar apex through the micropylar canal filled with secretions, (**E’**) at the cellular gametophyte central cell is visible, (**F**) mature ovule—pollen grains germinate on the surface of the nucellus when the prothallium contains mature archegonia and grow into the egg cell, (**F’**) the germinating pollen grain on the surface of the nucellus filled with secretions, (**G**) in vitro germinating pollen grain. *g* generative cell, *t* tube cell, *s* sterile cell, *p* prothallial cells, *nu* nucleus, *SC* sterile cell, *nu* nucleus, *in* integument, *st* stigmatic tip, *pst* pollinated stigmatic tip, *n* nucellus, *pg* pollen grain *t* tapetum, *M* megasporocyte, *DM* degenerating megaspores, *FM* functional megaspore, *FNG* free nuclear gametophyte, *ex* exine, *mc* micropylar canal, *v* vacuole, *CC* central cell, *CG* cellular gametophyte, *me* micropylar canal exudate, *EC* egg cell, *P* prothallium, *gp* germinating pollen grain, *ne* nucellus exudate, *dt* degenerating tapetum, *pt* pollen tube. Bars 10 µm.

The role of AGPs in the sexual reproduction of gymnosperms is not as well documented as that of angiosperms. Few studies have shown the presence of AGPs in the pollination drop [36] and the cell wall of the pollen grain and pollen tube of several species [37,38,39,40]. In our earlier work [41], we also identified and characterized the spatial and temporal distribution of AGPs during ovule differentiation in *Larix decidua* Mill. Here, we wanted to demonstrate the immunolocalization of AGPs in male gametophytes and ovules during their interaction. Similar to that in other gymnosperms, the processes of the pollination and germination of pollen grains in larch occurs in the different regions of the ovule. In *L. decidua*, the progamic phase is long and takes approximately two months. The pollen grains captured by the stigmatic tip of the ovule rest at the distal end of the micropylar canal and are then transferred upward to the nucellus apex. This transfer occurs after the canal is filled with secreted fluid. In the micropylar canal, pollen grains hydrate, shed their exine and germinate on the nucellus surface when the prothallium contains mature archegonia. Within a few days, the growing pollen tube penetrates the nucellus followed by neck cells and ventral canal cells and grows into the egg cell (Figure 1). This process differs from that noted in flowering plants, in which the stigma is both the site of pollination and germination of pollen grains and growing pollen tubes penetrate the pistil transmitting track to reach the embryo sac [1,42,43].

In *Larix*, two male gametes that form from the generative cell during pollen tube growth in the nucellus are discharged in the egg cell, and fertilization can occur. In the zygote second sperm cell and tube cell degenerate, while maternal and paternal organelles accumulate around the nucleus and are involved in the formation of the so-called neocytoplasm [41,44]. In angiosperms, the receptive synergid cell becomes the site of delivery of two sperm cells that participate in the process of double fertilization with egg cells and central cells, forming embryo and endosperm, respectively [45,46,47].

## 2. Results

### 2.1. Mature Pollen Grains and Unpollinated Ovule

**Figure 2:**
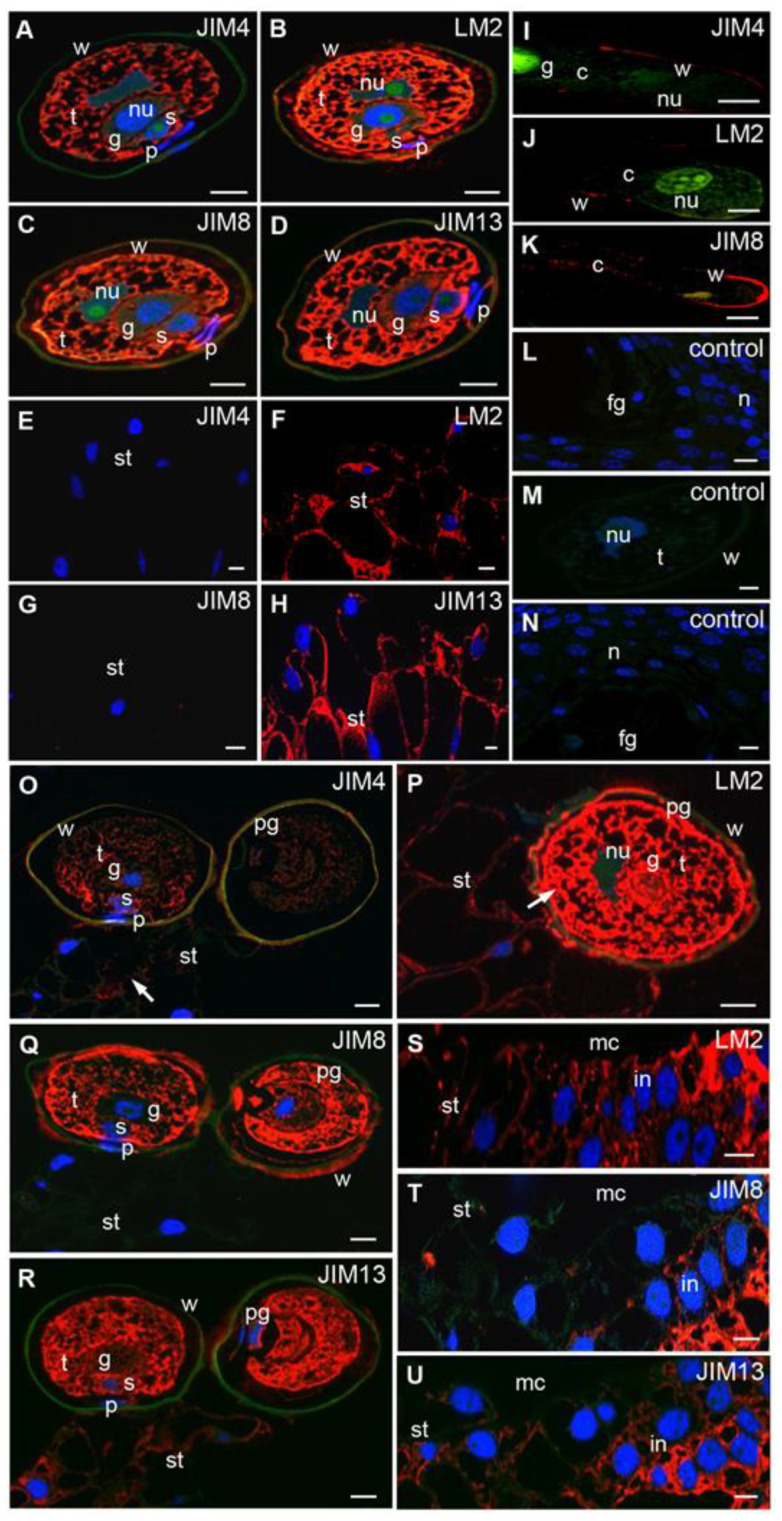
Immunolocalization of AGPs in larch male gametophytes and ovules. Mature pollen (**A**–**D**): JIM4 (**A**), LM2 (**B**), JIM8 (**C**,**D**) JIM13 Abs, all examined AGPs were present in the cytoplasm of all cells, weak signals of LM2, JIM8, and JIM13 Abs were visible in the pollen wall; (**E**–**H**) Unpollinated stigmatic tip: labeling with (**F**) LM2 and (**H**) JIM13 Abs was visible in the cytoplasm and in the cell wall, whereas (**E**) JIM4 and (**G**) JIM8 Abs were not detected; (**I**–**K**) in vitro growing pollen tube: JIM4 Abs were localized in the pollen tube wall (**I**), LM2 Abs were observed in the cell wall of aperture region (**J**), JIM8 Abs were detected at the tip of the pollen tube, only single spots of the fluorescence were also visible in the cytoplasm (**K**); (**L**–**N**) the control reactions: JIM13 Abs fluorescence was not observed in ovules preincubated with inhibitor-arabic gum prior to immunolocalization (**L**), no labeling was visible in the pollen grain when LM2 Abs were omitted (**M**) and the ovule which was not incubated with JIM13 Abs (**N**); (**O**–**R**) pollinated stigmatic tip: JIM4 Abs—the fluorescent signal was lower in the pollen compared with that noted before pollination, the low labeling appeared in the pollen wall and on the surface of the stigmatic tip (**O**), LM2 Abs—the higher signal in the wall and in the cytoplasm of the pollen was observed, whereas weak fluorescence was visible in stigmatic tip cells (**P**), JIM8 Abs were present in the pollen cells and on the surface of the pollen wall, the signal was not visible in the stigmatic tip cells (**Q**), JIM13 Abs—high fluorescence was detected in pollen cells, weak labeling in the cell wall and the cytoplasm of stigmatic tip cells was visible (**R**); (**S**–**U**) integument: LM2 Abs in the cells of stigmatic tip and integument (**S**), JIM8 Abs labeling was visible only in the integument cells below the stigmatic tip (**T**), JIM13 Abs—weak fluorescence in the stigmatic tip and the high level of the labeling in integument cells were observed (**U**). *g* generative cell, *t* tube cell, *s* sterile cell, *p* prothallial cell, *w* pollen/pollen tube wall, *pg* pollen grain, *fg* female gametophyte, *st* stigmatic tip, *mc* micropylar canal, *n* nucellus, *in* integument, *nu* nucleus, *c* cytoplasm, red—signal of the immocytochemical reaction, blue—DAPI staining, green—autofluorescence. Bars 10 µm.

In the mature, hydrated pollen grain, all examined AGPs were present in the cytoplasm of pollen cells (Figure 2A–D), excluding prothallial cells, where the JIM4 Abs signal was not observed (Figure 2A). In the pollen wall, single spots of analyzed epitopes in addition to JIM4 Abs were detected (Figure 2A–D). In epidermal and subepidermal cells of the unpollinated stigmatic tip, only AGPs recognized by LM2 (Figure 2F) and JIM13 (Figure 2H) Abs were visible. The labeling was present in the cell wall and the cytoplasm of these cells.

### 2.2. The Pollination Stage

In pollen grains adhering to the stigmatic tip, all examined AGPs were mostly present in the tube cell cytoplasm (Figure 2O–R). Interestingly, pollination induced a reduction in JIM4 Abs labeling in pollen cells and the appearance of a weak signal in the pollen wall (Figure 2O). The patterns of LM2 (Figure 2P), JIM8 (Figure 2Q) and JIM13 (Figure 2R) Abs distribution in all pollen cells were similar to those observed in these cells before pollination. Only in the pollen wall the increase in the signals of AGPs recognized by LM2 (Figure 2P) and JIM8 Abs (Figure 2Q) was detected.

Immediately after pollination in the stigmatic tip in the region of pollen grain adhesion, weak fluorescence derived from JIM4 Abs appeared (Figure 2O). LM2 (Figure 2P) and JIM13 (Figure 2R) Abs labeling was reduced compared with that noted before pollination. Epitopes detected with LM2 Abs were exclusively observed in the apoplast of the stigmatic cells, while the cytoplasm of these cells was not labeled. The highest level of these AGPs was visible at the surface of the stigmatic tip exactly at the site of pollen adhesion (Figure 2P). Before pollination, the epitopes recognized by the JIM8 Abs were not detected in these cells (Figure 2Q). At this stage of ovule development, strong fluorescence of LM2 (Figure 2S), JIM8 (Figure 2T) and JIM13 (Figure 2U) Abs was visible in the integument cells, while the epitopes recognized by JIM4 Abs were not detected.

### 2.3. Pollen Grains in the Micropylar Canal

**Figure 3:**
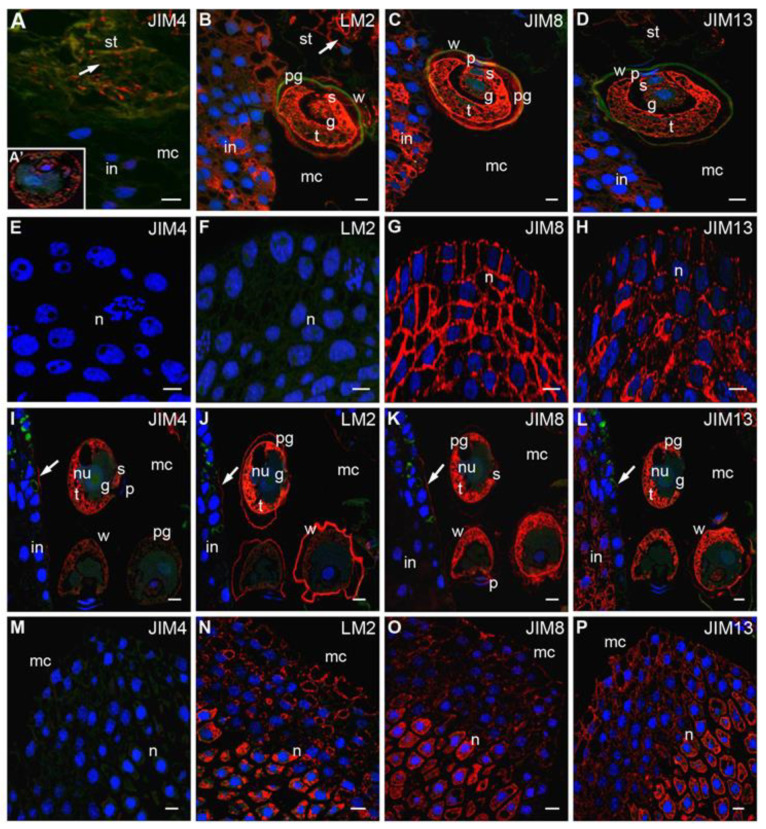
Immunolocalization of AGPs in larch pollinated ovules. (**A**–**H**) The pollen in the micropylar canal. (**A**–**D**) Micropylar canal: The epitopes recognized by JIM4 Abs occurred in the material that formed as a result of the degeneration of the stigmatic tip cells, while labeling was not observed in the integument (**A**), the fluorescent signal of JIM4 Abs in pollen grain was also visible (**A’**), LM2 Abs—the signal was present in the cytoplasm and the pollen wall and stigmatic tip cells (arrow), the fluorescence was present mainly in integument cell cytoplasm, the region of pollen grain adhesion and the apoplast (**B**), JIM8 (**C**) and JIM13 (**D**) Abs—labeling was present in the pollen grain and in the integument cells away from the region of the pollen adhesion; (**E**–**H**) Nucellus: cells were completely devoid of the fluorescence from JIM4 (**E**) and LM2 (**F**) Abs, only JIM8 (**G**) and JM13 (**H**) Abs were observed; (**I**–**P**) pollen grain in the micropylar canal. All studied AGPs occurred in the cytoplasm of the pollen grain—JIM4 (**I**), LM2 (**J**), JIM8 (**K**), JIM13 (**L**), only LM2 Abs was visible in the pollen wall (**J**), small clusters of signal from examined Abs were also present in the surface of the micropylar canal (**J**, arrow), only JIM13 Abs labeling was localized in the subepidermal cells in the integument (**L**); (**M**–**P**) Nucellus: JIM4 Abs labeling was not visible (**M**), LM2 (**N**), JIM8 (**O**) and JIM13 (**P**) Abs were widely localized in the ecm in the apical region of the nucellus and the central area of this tissue in the cytoplasm. *g* generative cell, *t* tube cell, *s* sterile cell, *p* prothallial cell, *w* pollen wall, *pg* pollen grain, *st* stigmatic tip, *mc* micropylar canal, *n* nucellus, *in* integument, *nu* nucleus. red—signal of the immocytochemical reaction, blue—DAPI staining, green—autofluorescence. Bars 10 µm.

At the time of pollen grain engulfment into the micropylar canal, the strong signals after labeling with LM2 (Figure 3B), JIM8 (Figure 3C) and JIM13 (Figure 3D) Abs remained present in the pollen cells cytoplasm, while weak fluorescence was visible in the pollen wall. JIM4-reactive AGPs were localized at low levels in the cytoplasm of pollen cells (Figure 3A’), and the material formed as a result of stigmatic tip degeneration. In the apoplast of stigmatic tip cells, epitopes recognized by LM2 (Figure 3B) and JIM13 (Figure 3D) but not JIM8 (Figure 3C) Abs were present. However, in the integument, cells were still labeled by all three Abs (Figure 3B–D). The fluorescence signal from these Abs was clearly reduced in the cells adjacent to the pollen grain. JIM4 Abs labeling was noted in small single spots of fluorescence in the ecm of stigmatic tip cells, while this fraction of the AGPs was not detected in the integument cells (Figure 3A).

In the nucellus, only the fluorescence derived from both JIM8 (Figure 3G) and JIM13 (Figure 3H) Abs was observed. In epidermis and subepidermis cells, the signal was localized in the apoplast. The signal was also localized in the cytoplasm in the cells situated deeper. In this stage, no labeling by JIM4 (Figure 3E) or LM2 (Figure 3F) Abs was detected in nucellus surface cells.

During transfer to the nucellus, pollen grains shed their exine, and crucial changes in the localization of AGPs epitopes were observed. The distribution of JIM4 (Figure 3I) and LM2 (Figure 3J) Abs varied in different pollen grains. Some pollen grains were almost completely devoid of labeling, whereas intense fluorescence was detected in the tube and sterile cells in other pollen grains. Comparable levels of signals from JIM8 and JIM13 Abs were noted in all pollen grains. Interestingly, none of the examined epitopes were observed in generative cells (Figure 3I–L). In the pollen wall, only a strong signal of LM2 Abs labeling was detected (Figure 3J), while the signals of JIM4 (Figure 3I), JIM8 (Figure 3K) and JIM13 (Figure 3L) Abs were weak.

During this period, considerable changes in the distribution of different AGPs were observed in the integument. In the wall coating micropylar canal, AGPs recognized by JIM4 Abs appeared (Figure 3I), while LM2 (Figure 3J) and JIM8 (Figure 3K) Abs labeling substantially decreased in all integument cells. The weak fluorescence signal was visible only on the surface of the micropylar canal. The epitopes recognized by the JIM13 Abs were particularly localized in the subepidermal cells of the integument, while the wall coating micropylar canal was almost completely devoid of labeling (Figure 3L).

When pollen grains engulfed the micropylar canal, changes in the distribution of AGP epitopes were also observed in the nucellus. As previously noted, JIM4 Abs labeling was not detected (Figure 3M), while nucellus cells were still labeled with the JIM8 (Figure 3O) and JIM13 (Figure 3P) Abs. Fluorescence indicating LM2 Abs detection also appeared. The distribution of these AGPs epitopes was comparable. On the surface layer of the nucellus, the signal was localized in the apoplast. However, the signal was also localized in the cytoplasm in cells situated in the central region of the nucellus.

### 2.4. Pollen Grain Germination and Pollen Tube Growth

**Figure 4:**
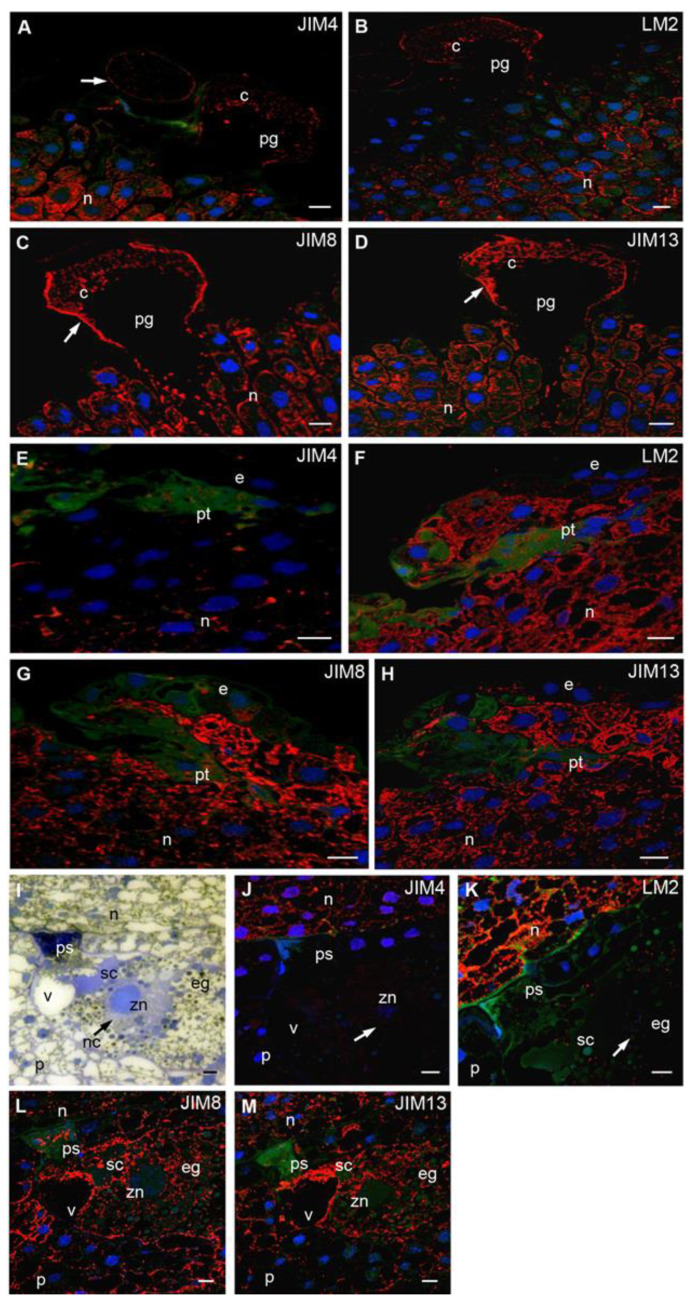
Immunolocalization of AGPs in larch ovules during pollen germination and pollen tube growth. (**A**–**D**) Nucellus with germinating pollen: all examined AGPs occurred in the cytoplasm and wall (arrow) of the germinating pollen—JIM4 (**A**), LM2 (**B**), JIM8 (**C**), JIM13 (**D**) Abs, in the nucellus JIM4 (**A**) and LM2 (**B**) Abs were present in the cells localized under its surface, whereas JIM8 (**C**) and JIM13 (**D**) Abs were visible in the cytoplasm and in the ecm of cells surrounding the germinating pollen tube; (**E**–**H**) Pollen tube growth: the cells adjacent the growing pollen tube were almost devoid of JIM4 (**E**) Abs labeling, whereas LM2 (**F**), JIM8 (**G**) and JIM13 (**H**) Abs were present in the cytoplasm as well as in the ecm of these cells; (**I**–**M**) ovule after fertilization: toluidine blue staining, in the micropylar region of the fertilized egg cell, at the site of the pollen tube entry the polysaccharide plug was visible, the zygote nucleus was surrounded by neocytoplasm (arrow), in the egg cell second sperm cell was also present (**I**); (**J**–**M**) Immunolocalization of AGPs in the fertilized ovule: JIM4 Abs—the prothallium and fertilized egg cell were devoid of the labeling, the signal was only localized in the nucellus (**J**); LM2 Abs—a strong signal in the nucellus apoplast and numerous foci of fluorescence in the perinuclear area of egg cell were observed (**K**). JIM8 (**L**) and JIM13 (**M**) Abs labeling was visible in the fertilized egg cell and the apoplast of the nucellus as well as the prothallium. *e* epidermis, *eg* egg cell, *n* nucellus, *nc* neocytoplasm, *p* prothallium, *pg* pollen grain, *ps* polysaccharide plug, *pt* pollen tube, *sc* sperm cell, *v* vacuole, *zn* zygote nucleus. red—signal of the immocytochemical reaction, blue—DAPI staining, green—autofluorescence. Bars 10 µm.

Pollen grain germination and pollen tube growth are periods of archegonia maturity. At this time, all studied AGPs were detected in the cytoplasm of tube cell in the pollen grain (Figure 4A–D). The signals of all Abs were also clearly visible on the surface of the pollen. Nucellus cells localized in the region of the germinating pollen grain were weakly labeled with JIM4 (Figure 4A) and LM2 (Figure 4B) Abs in contrast to the cells situated below the nucellus apex, in which a strong signal was observed. The JIM4 Abs (Figure 4A) were distributed evenly in the cytoplasm, while the LM2 Abs (Figure 4B) were mainly localized in the apoplast of these cells. Epitopes recognized by JIM8 (Figure 4C) and JIM13 Abs (Figure 4D) were present in all nucellus cells and adjacent to the germinating pollen grain. The fluorescence signal was visible both in the cytoplasm and in the ecm.

In nucellus cells surrounding the growing pollen tube, only single spots of fluorescence from JIM4 Abs (Figure 4E) were observed in contrast to the LM2 (Figure 4F), JIM8 (Figure 4G) and JIM13 Abs (Figure 4H), in which increased signal was noted in the cytoplasm and apoplast. At this time, the fluorescence intensities of all examined Abs were clearly reduced only in the epidermal cells.

### 2.5. Fertlization

In *L. decidua,* the pattern of the immunocytochemical localization of examined epitopes of AGPs in the archegonium and the egg cell before fertilization was described previously by Rafińska et al. [41]. In this work, we focused on the last stage of the interaction between male gametophyte and ovule when the pollen tube penetrates archegonium and grows into the egg cell. In the micropylar region of the egg cell at the site of pollen tube entry, a polysaccharide plug was visible (Figure 4I). Fertilized egg cells also contain large vacuole and sperm cells that do not participate in the process of fertilization. At this time, the zygotic nucleus is surrounded by the neocytoplasm. In the zygote epitopes recognized by JIM4 Abs (Figure 4J), no labeling was detected; however, the labeling of JIM8 (Figure 4L) and JIM13 (Figure 4M) Abs was intense. LM2 Abs were localized either as single (Figure 4K) or numerous (Figure 4K’) clusters of fluorescence, especially around the perinuclear space. Spots of the fluorescence of JIM8 Abs (Figure 4L) were also found in the polysaccharide plug. After fertilization, all studied AGPs were still localized in the ecm of the nucellus cells (Figure 4J–M).

### 2.6. In Vitro Growing Pollen Tubes

Using light microscopy, we could not confirm whether the observed localization of the studied AGPs epitopes was present in the wall of growing in vivo pollen tubes or only in the ecm of nucellus. Thus, we assessed AGPs in pollen tubes growing in vitro (Figure 2I–K). Our results revealed that epitopes binding JIM4 (Figure 2I) and JIM8 Abs (Figure 2K) were localized throughout the pollen tube wall, while epitopes binding by LM2 Abs (Figure 2J) were limited to the cell wall in the aperture region (Figure 2J). The accumulation of JIM8 Abs signals was observed in the tip of the growing pollen tube, and a small signal was also visible in the cytoplasm (Figure 2K). AGPs recognized by JIM13 Abs were not identified.

### 2.7. Control Reactions

Control sections incubated in the absence of primary antibody or with a mixture of JIM13 Abs with the corresponding inhibitor were devoid of immunolabeling (Figure 2L–N).

**Figure 5:**
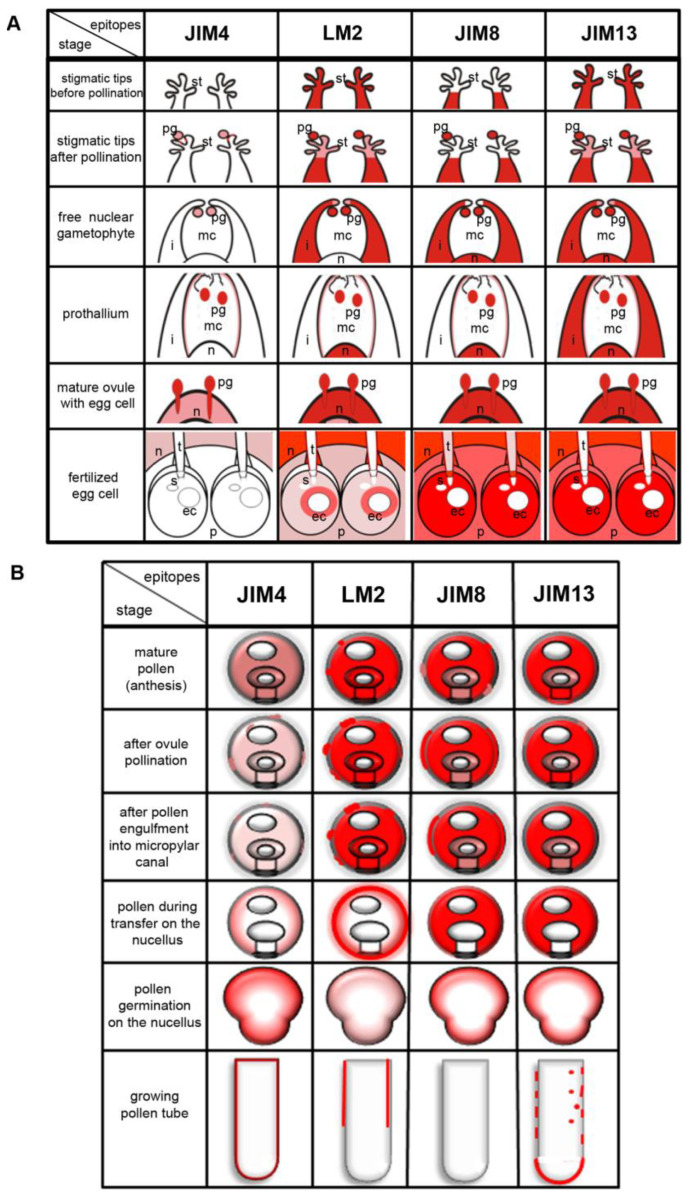
Schematic localization of AGPs (red colour) (**A**) during successive stages of male gametophyte and ovule interactions and (**B**) in vivo developing male gametophyte and in vitro growing tube of *L. decidua*.

Temporal and spatial changes in the distribution of the different categories of AGPs during male gametophyte–ovule interaction in *L. decidua* are summarized in Figure 5.

## 3. Discussion

In mature pollen, all examined AGPs were present in the cells’ cytoplasm. The pollen wall was devoid of the AGPs recognized by JIM4 Abs, and only a small pool of epitopes binding LM2, JIM8 and JIM13 Abs was detected. Additionally, the cells of the unpollinated stigmatic tip were deprived of AGPs binding JIM4 Abs, and only AGPs recognized by LM2 and JIM13 Abs were observed. Pollination induced a significant qualitative change in the distribution of AGPs in the pollen wall and stigmatic tip cells. In the pollen grain, AGPs were secreted from the cytoplasm to the sporoderm. This AGPs pool may participate in the adhesion of pollen to the ovule. Additionally, in the region of physical contact between the stigmatic cells and the pollen wall, AGPs bound by JIM4 Abs appeared. It is possible that, in *Larix*, the appearance of AGPs recognized by JIM4 Abs in stigmatic cells and the pollen wall may reflect the acquisition of receptivity by the ovule and play an important role during the early interaction steps between the reproductive partners. In stigma cells of angiosperms, AGPs [2,48,49,50] are responsible for the adhesion of pollen grains [51]. Sage et al. [50] postulate that AGPs and HGs form a hydrophilic network that allows the transport of water and molecules responsible for the interaction of the pollen grain and stigma. Thus, we suggest that in *Larix*, AGPs may exhibit a similar function [41].

Significant changes in the distribution of AGPs in pollen grains occur during their transfer to the nucellus apex. Exine rupture and shedding occur during this period. AGPs secreted from pollen, especially those labeled by LM2 Abs, can be associated with pollen wall hydration and swelling. During pollen grain hydration in *N. tabacum* and *Lycopersion peruvianum,* AGPs secretion was also observed [52]. Moreover, in *A. thaliana,* AGPs participate not only in the control of pollen grain hydration but also at the time of germination [53]. In the double mutant, *agp6* and *agp11* pollen grains germinate in the anther, indicating that these categories of AGPs prevent the premature growth of pollen tubes. In light of these data, we suggest that the precise control of these two processes in *Larix* seems to be very important because pollen hydration and germination are separated in “time and space” and must be synchronized with female gametophyte development. However, the participation of AGPs in these processes requires further study.

In *L. decidua,* during pollen grain movement in the micropylar canal upward to the nucellus before they germinate to the pollen tubes, a small pool of studied AGPs, including AGPs recognized by JIM4 Abs, is secreted from the epidermal cells onto the inner surface of the integument. Initially, these AGPs were located near the pollen grains and then into the cells of the entire structure. Immediately before fluid secretion, the integument surface lining the micropylar canal is further coated by a hydrophilic membrane in *L. eurolepsis* [54]. This integumentary membrane of the micropylar canal of *Pseudotsuga* is created from membrane portions of vesicles formed in the cytoplasm of inner epidermal cells of the integument that traverse through the cell wall and release their contents on its surface [55]. In gymnosperms, the presence of the integumentary membrane may be an element of the mechanism responsible for pollen transfer to the nucellus apex [54]. In the *Larix* fluid secreted in the micropylar canal examined, AGPs fractions were not found. This finding indicates that either they are not secreted into the micropylar canal or are undergoing such modifications that they cannot be bound by the anti-AGPs Abs used. In contrast to *Taxus x media,* the epitopes recognized by LM2 and JIM3 Abs were localized in pollination drop [36]. In the future, we will perform more precise experiments in *Larix* to verify this hypothesis.

During the germination of pollen grains, AGPs recognized by JIM4 Abs appeared for the first time in the cytoplasm followed by the apoplast of the nucellus cells. It is possible that this specific category of AGPs is a marker of the next stage of ovule development and reflects the preparation of the nucellus/archegonium to receive pollen tubes. Then, during pollen tube growth, this fraction of AGPs is metabolized, as evidenced by their decreasing levels. During the germination of pollen grains AGPs bound by LM2, JIM8 and JIM13 Abs were still present in the cytoplasm of the nucellus cells. When the pollen tubes grew in the nucellus, the pool of AGPs recognized by LM2 Abs increased, and all fractions of AGPs were secreted to the apoplast, where they directly physically interacted with male gametophyte. A similar distribution of AGPs was observed in the ecm of the nucellus cells of *T. x media*, where AGPs were bound by LM2 and JIM13 Abs [36]. In *Larix*, it is possible that AGPs present in the apoplast of the nucellus become the part of secretion and may act as adhesive, nutrient or chemotactic molecules. Numerous studies performed in angiosperms show that AGPs are commonly found on the stigma and in the transmission tissue of the pistil, where ecm is the site of adhesion and germination of pollen and pollen tube growth. The specific accumulation of AGPs recognized by LM2 Abs and its secretion into the apoplast of nucellus may indicate that this fraction of AGPs in larch may be related to pollen tube reception by the mature receptive gametophyte so that it can grow directly into an egg cell, which is similar to that noted in flowering plants [33,56,57,58,59].

After fertilization in the cytoplasm of the egg cell, the high pool AGPs recognized by JIM8 and JIM13 Abs were localized, while AGPs bound to JIM4 Abs were not detected. We observed a similar pattern of localization of these AGPs in the cytoplasm of egg cell before fertilization [41]. It is possible that these fractions of AGPs can be markers of material designed for degradation, e.g., from the pollen tube, and participate in the reorganization of the egg cytoplasm [41]. In *A. thaliana,* AGP4, which is also named JAGGER, is a cell death marker for persistent synergid degeneration and polytubey block [60,61]. Thus, AGPs recognized by LM2 Abs localized around the zygote nucleus may be involved in the organization of the neocytoplasm. During this process, around the condensing nucleus of the fertilized egg cell, the accumulation of the organelles of the pre-embryo was observed [41].

The role of AGPs in early plant embryogenesis is known and has been described in both zygotic and somatic embryogenesis [62,63,64,65,66]. AGPs participate in embryo development and nutrient absorption and may also be involved in the formation of a new cell wall during cell division [63,66,67,68]. The addition of purified AGPs to the culture of embryogenic cells, stimulated or inhibited somatic embryogenesis in the gymnosperm *Picea abies* depending on the fraction of AGPs [69,70].

Studies with Yariv reagent have revealed the effect of AGPs on pollen tube growth [71]. AGPs induce cell wall modification by altering the localization of cellulose and HGs and the secretion of callose [56,72]. These modifications play a significant role in the mechanical properties of the pollen tube wall [73]. In *L. decidua,* we suggest that AGPs recognized by LM2 Abs localized in in vitro growing pollen tubes only in the germinal aperture may play an important role during pollen germination and tip formation. Thus, pollen tube tip elongation and signaling from female gametophyte are likely mainly related to AGPs recognized by JIM8 Abs. This pool of AGPs was localized in the wall and cytoplasm of in vitro growing pollen tubes, especially in the tube apex. In male gametophytes, JIM8 Abs were specifically localized in the ecm of neck cells through which the pollen tube grew into egg cells [41]. Costa et al. [20] proposed that, in *A. thaliana*, AGP6/AGP11 may be involved in signaling, endocytosis (recycling) and secretion processes in the pollen tube apical dome during pollen tube growth, promoting the remodeling of the plasma membrane and maintaining the loosened wall essential for growth. In turn, in the subapical zone, HG cross-linking calcium impedes AGPs contact with signal molecules, making AGPs inactive [74]. The proposed model for the signaling role of AGPs during pollen tube tip growth is very attractive, and future studies will address this possibility in *L. decidua*.

In in vitro growing pollen tubes of *Larix*, we did not detect AGPs labeling by JIM13 Abs; however, this category of proteins was present in the cytoplasm of mature pollen grains. A similar localization of this fraction of AGPs was observed in *Nicotiana alata*. In this species, AGPs bound by MAC207, JIM8 and JIM13 Abs were also noted in pollen grains, but their presence was not revealed in growing pollen tubes [52]. JIM13 Abs labeling was not detected in pollen tubes of *A. thaliana* [75,76].

## 4. Materials and Methods

### 4.1. Sample Preparations

Female cones of *Larix decidua* Mill. were collected from March to June twice a week from trees growing in the garden of the Faculty of Biological and Veterinary Sciences, Nicolaus Copernicus University, Torun, Poland. Based on the correspondence between the male–female gametophytes, the ovules were isolated at the successive stages of development: (1) megasporocyte—period of the pollen shed, (2) the functional megaspore—stigmatic tip of the ovule is pollinated, (3) free nuclear stage—pollen grains are engulfed into the micropylar canal of the ovule, (4) cellular stage—pollen grains are carried to the nucellar apex, (5) mature ovule—pollen tubes penetrate the nucellus [44]. Dissected ovules were immediately fixed in 4% paraformaldehyde and 0.25% glutaraldehyde (Sigma, St. Louis, MO, USA) in PBS pH 7.2 for 24 h in 4 °C and then the material was dehydrated in a graded ethanol series containing 10 mM dithiothreitol (DTT, Thermo Fisher Scientific, Waltham, MA, USA) and embedded in BMM resin (butyl methacrylate, methyl methacrylate) with 0.5% benzyl ethyl ether (Sigma, St. Louis, MO, USA) and 10 mM DTT (Thermo Fisher Scientific, Waltham, MA, USA) at −20 °C under UV light for polymerization. Then, the ovules were cut on a Leica UCT ultramicrotome (Leica, Wetzlar, Germany) into semithin section (1 µm) and placed on microscope slides coated with biobond (British Biocell International, Cardiff, UK). Before performing immunocytochemical reactions, the BMM resin was removed by washing the sections in pure acetone twice for 10 min and then in water and, finally, in PBS pH 7.2 [7,44,77]. 

Mature male cones were collected in March and April. Next, they were sterilized in 70% ethanol for 40 s and in 10% sodium hypochloride for 45 s. The material was rinsed in distilled water and then dried in sterile Petri dishes covered with sterile filter paper. Pollen grains were stored for approximately 2 months until the ovules matured. Before culturing, pollen grains were hydrated for 24 h at 24 °C in sterile condition and then placed in culture medium contained Brewbaker and Kwack minerals diluted in 1:10 supplemented with 18% PG 4000, 7% sucrose, 0.4% phytagel, nystatin (0.0041 g/25 mL) and chloramphenicol (0.0014 g/25 mL), and the pH was adjusted to 5.2. Additionally, sterilized nucelli isolated from mature female cones were added to the cultures. The cultivation was carried out for 7–14 days in the dark. Next, pollen tubes were fixed in 4% paraformaldehyde and 0.25% glutaraldehyde (Sigma, St. Louis, MO, USA) in PBS pH 7.2 for 2 h at RT and then individually transferred to cover glasses coated with a drop of poly-l-lysine (Sigma, St. Louis, MO, USA) [44]. 

### 4.2. Immunolocalization Experiments

Semithin serial section of the ovules and in vitro germinating pollen grains and pollen tubes after blocking with 0.5% BSA (Sigma, St. Louis, MO, USA) in PBS pH 7.2 for 10 min at RT were incubated with the primary Abs diluted 1:50 in 0.1% BSA in PBS pH 7.2 overnight at 4 °C. We used anti-AGP LM2, JIM4, JIM8 and JIM13 Abs listed in Table 1. After washing with PBS pH 7.2, signals were detected using secondary anti-rat IgG Abs that was conjugated with Cy3 (Jackson ImmunoResearch Laboratories, West Baltimore Pike, PA, USA) diluted 1:1000 in 0.2% BSA (Sigma, St. Louis, MO, USA) in PBS pH 7.2 for 1 h at RT in the dark. After washing with PBS pH 7.2 DNA was stained with DAPI (Sigma, St. Louis, MO, USA). Then, the sections were washed in distilled water, dried at RT and covered with 0.5% *w/v* N-phenylenediamine (Sigma, St. Louis, MO, USA). A control was performed without primary antibody, or JIM13 Abs were preincubated with the specific inhibitor-arabic gum (Sigma, St. Louis, MO, USA) for 1 h prior to immunolocalization [78].

Semithin sections were observed in a Nikon Eclipse 80i fluorescence microscope equipped with a Plan Fluor 40× (numerical aperture, 0.75) lens and narrow band filters (UV-2EC, B-2EC, G-2EC) (Nikon Europe BV, Badhoevedorp, The Netherlands). Results were photographed using a Nikon DS-5Mc color cooled digital camera and Lucia G 5.30 image analysis software. Camera settings were kept constant for exposure time, gain and offset. In turn in vitro growing pollen tubes were analyzed with Nikon Eclipse TE300 confocal laser scanning microscope using an argon-ion laser emitting light with a wavelength of 488 nm (autofluorescence) and helium-neon laser emitting light with wavelength of 543 nm (Cy3). A 100× (N.A. 1.3) Plan Fluor H/N2 oil immersion lens was used. The EZ 2000 Viewer software package for image processing and analysis was used (Nikon Europe BV, Badhoevedorp, The Netherlands) [44].

## 5. Conclusions

Taken together, our studies reveal that in *L. decidua*, AGPs are involved in the interaction between the male gametophyte and the ovule:

1. A special role in these processes is that AGPs recognized by JIM4 Abs always appear in the ovule in the contact region with the pollen grain or the pollen tube, i.e., in the pollinated stigmatic tip cells during the transfer of the pollen grain to the nucellar apex and in the nucellus overgrown by the pollen tubes.
2. AGPs accumulate in the cytoplasm of mature pollen and are secreted into the pollen wall after pollination, probably participating in their adhesion to the ovule.
3. AGPs recognized by LM2 Abs are probably involved in the hydration and germination of pollen grains, while AGPs labeled by JIM8 Abs are essential for pollen tube tip growth and signaling with the female gametophyte.
4. The distribution of AGPs in the nucellus reflects its preparation for interactions with pollen tubes. AGPs are synthesized and stored in the cytoplasm of nucellus cells. During pollen tube growth, these molecules are secreted into the ecm, where they can directly interact with the male gametophyte.
5. AGPs recognized by LM2 Abs are likely to participate in the reorganization of the cytoplasm of fertilized egg cells.

## Figures and Tables

**Table 1:** Antibodies used to detect AGPs epitopes.

Antibody	Epitopes	References
JIM4	β-d-Glc*p*A-(1→3)-α-d-Gal*p*A-(1→2)-l-Rha and possibly others unknown data	[79]
LM2	β-d-Glc*p*A, β-d-Glc*p*A(1-*O*-Me) and possibly other unknown data	[80]
JIM8	Carbohydrate portion of AGP	[81]
JIM13	β-d-Glc*p*A-(1→3)-α-d-Gal*p*A-(1→2)-l-Rha and possibly other unknown	[79]

## Data Availability

Not applicable.
